# The Bloodletting Question in Olden Times

**Published:** 1859-01

**Authors:** Thomas K. Chambers

**Affiliations:** Physician to St. Mary’s Hospital


*The Bloodletting Question in Olden Times.* By THOMAS K. CHAMBERS, M.D., Physician to St. Mary's Hospital.

Now that the question of the propriety of bloodletting in the treatment of disease seems again likely to invite public attention, it may be amusing to our readers, and perhaps not unprofitable, to reflect on the way in which the same subject affected men's mind seventeen centuries ago. It is the duty of an historian to avoid partisanship, and not always wise for him to prophesy; still, it must be remarked that the danger to which we are now drifting seems to be not dissimilar from the prejudices which Galen then found prevalent in Rome. The risk to a patient now is, not as twenty years ago, that he will be bled unnecessarily, but that bleeding will be abstained from when really requisite. Human nature does not alter, though some fallacies leading perhaps to strangely different results enter into our minds through the same portals as they did into our forefathers; and the record of the past, rightly interpreted, cannot but teach wisdom for the future. Let us then fancy ourselves in the metropolis of the world, prosperous and glorious under the rule of the Antonines, in the latter half of the second century, and let us hear Claudius Galen lecture the public in his pleasant chatty style 'Against certain Erasistrateans' :*

"When I first came to Rome† I found some physicians who were so averse to venesction, that sometimes, when a man was scarce able to breathe from congestion, they would not employ this treatment.—There was a woman, just under twenty-one, who after suppression of the catamenia, had a flushed face, with loose cough and dyspnœa, whom they treated by bandaging the limbs and depriving her entirely of food; but they would neither open a vein nor let me do so. And on account of their being acquaintances of the woman's household and senior practitioners, more faith was had in their opinion than in mine. I made no more attempts to persuade them to bleed, but I asked if there was any objection to set up a derivation of blood towards the uterus by means of drugs calculated for that object. And when they consented, I immediately got the midwife usually employed by the patient, and desired her to use them. But she said she had already applied remedies of this sort at the proper time—namely, when the cata menia might normally be expected; and she named the drugs—all of

* Galeni Opera, Edit. Kuhn, vol. xi. p. 187.

† Viz, in the thirty-fourth year of his age, A. D. 165.

tried efficacy—which she had administered to the woman, so that no one could suppose that it was from the inefficiency of these medicines that relief had failed to be given. When I heard this, and moreover that the menses had been already suppressed four months, I had another consultation with the medical men to try and persuade them to bleed. When they refused I wondered why, if they were anxious to evacuate the superfluous blood through the uterus by opening the mouths of the veins there, yet they should think the evacuation injurious when it was made by opening any other vein. They stated that 'superfluous blood could be evacuated by fasting alone,' without having recourse to treatment such as I proposed. So I held my tongue and took my leave, in despair about the woman, on account of the cough and dyspnœa. I expected that she would either spit blood from the chest, or from the lungs by the bursting of a bloodvessel, or would have laryngitis, or pleurisy, or pneumonia; and my hope was, as a choice of evils, that she would have pleurisy for I was afraid, in case of laryngitis and pneumonia, that the risk would be imminent, and that in case of hæmoptysis, that the occurrence of it would be fatal. And such turned out to be the result. For as she was coughing very violently, blood was thrown up. And now some non-professional persons complained of the doctors who opposed the bleeding; and hopes were expressed that now at least, though not before, they would be shamed into permitting the treatment. When they would not give way, but desired the bandages round the limbs to be tightened, and persisted in the attempt at derivation towards the uterus, and in continuing the starvation, I took my leave persuaded that I could effect nothing on account of the gentlemen's age and celebrity. And very shortly afterwards the patient was seized with an incurable difficulty of breathing, and died.

"Under the hands of the same physicians who opposed bleeding there also died several patients with laryngitis. And there was another patient, too, who through the whole winter had been living high, and taking no exercise, and in the spring was as red in the eyes and face as a man kept for a long time with his head on the ground and his legs in the air, and he died suffocated after five days' illness.

"Next there was a fourth patient—a woman—who was ill at the same time that the catamenia were suddenly stopped, whom these enemies to bleeding brought to death's door. They kept her for three days absolutely without food, because she had a continued fever; on the fourth day they gave her the smallest possible quantity of slops; on the fifth day they ordered fasting again, and then she got violently delirious, jumped up, and ran screaming about out of doors, and the attendants had great difficulty in restraining her violence. She, however, was saved by nature, through a copious effusion of blood from the nostrils.

"This was a circumstance that should excite our admiration, and at the same time teach us what a powerful influence bloodletting has in such affections, for immediately after the hæmorrhage from the nostrils the woman was freed from all her symptoms.

"Now previously to this I had shunned having any communication with the medical men, guessing what they would say against the use of venesection. But since it was so very clear to all that the woman's life was saved by the evacuation of the blood, I recalled to their memory the fatal cases, expressing an opinion that perhaps those, too, would have been saved if they had been bled. And I gave sundry reasons for it. But these gentlemen involved the matter in a maze of words, twisting the argument round and round, and up and down, and came to no conclusion. However, they at last ended by taking refuge in Erasistratus, stating that it was 'shown by him in his First Book on Loss of Blood that it was better to apply ligatures to the limbs than to bleed.'"

Here is the matter brought. The "blood-funkers" with an absence of originality truly Roman, had no reason for refusing to bleed beyond the opinion against it of Erasistratus, who had been dead four hundred years. Strongly in accordance with human feelings must his teaching have been to have prevailed so long in opposition to that of Hippocrates and his successors! What could his grounds have been? In the first place, he was an anatomist, and a morbid anatomist; and having observed in the bodies of those who died of febrile affections the arteries congested with black venous blood, whereas in animals or men killed in health they were empty, he thought the cause of fever was the blood getting from the veins into the arteries. So, argued he, the best way of treating the disease is to keep the blood in the veins; and he tried to effect this object by the expedient of putting ligatures round the limbs. Secondly, he looked to local congestion as the originating cause of all diseases,* as well as those commonly called idio-

* Erasistratus was one of the founders of the great anatomical school of Alexandria in the third century before Christ. He first traced the origin of the veins to the heart, named the tricuspid valves, and assigned to them their true office, described the lacteals, distinguished the nerves of motion and sensation, and was bold enough to confess he could find no use for the spleen. He was a skilful operator, and invented a sound called for a long time after his name.

pathic as others, and consequently that the treatment of both must be the same. Seeing, then, before his eyes (we may suppose) low fever get well the quicker without bleeding, his theory led him to conclude the same of all fevers, even when accompanied or caused by obvious local inflammation. Thirdly, he was a pupil of Chrysippus, who was a devoted Pythagorean, with religious scruples, probably of Egyptian origin, against shedding of blood, which scruples his shrewd courtly pupil* replaced by physiological arguments. Fourthly, seeing that the blood was formed out of the food, he conceived that withholding nutriment was quite as sure, at the same time that it was a safer, way of diminishing the amount of circulation than venesection. He therefore adopted the practice, common enough in France still, though not practiced in Britain, of "*diete absolue.*"

Considering what we owe to Erasistratus as a physiologist, one feels grieved at being obliged to extract his arguments secondhanded from the works of such a vigorous opponent as Galen. The above, however, seems to be a fair statement of the mode of deduction by which he arrived at a conclusion influencing practice so many hundred years after his death as Galen's time, and at a place so distant as Rome. But stay—before we wonder at their power, are we quite sure that closely analogous influences do not weigh with us now?

In the first place, are we not often too much governed by theoretical explanations of what is seen in the body? Are we not too apt to forget the difference between that flabby decomposing substance and the brimming whirl of life, such as we see it faintly pictured in a frog's web under the microscope?

Secondly. Do we not still see confounded into one common mass, and treated with one treatment, cases of low and cases of plegmonous inflammation! We see, e.g., statistical tables of pneumonia, typhous and croupous together, where *all*, old, young, and middle-aged, have

* One instance of the *savoir faire* of Erasistratus is amusing. He was physcian to Seleucus Nicator, King of Syria, and was consulted by him about the heir-apparent, Antiochos. He finds that the young man's complaint is love, and that the beloved object is one who it will be easily understood would create no slight commotion at court when known. So he informs the sovereign mysteriously of the nature of the difficulty, saying that the poor youth will probably die unless he possesses the object of his affections, and that she is no other than Mrs. Erasistratus. His majesty, thinking, like the public in general, that medical men are their absolute property, begs the doctor to oblige him but this once, and save his heir's life by the gift of the lady. Then does our shrewd prophet interpret his parable—"*thou* art the man; it is *your* queen Stratonice that is the only available prescription." Strange to say, the king was unpractical enough to be persuaded by such an *ad hominem* argument.—Biographie Universelle, Article "Erasistrate."

been bled, and antagonistic tables where *none* have been bled; and, as one might expect, the results are often numerically the same. Will not some one make a table of cases where those were bled who *ought* to have been, and where those were not bled who *ought not* to have been? It may be safely predicted, that a much larger percentage of recoveries will be exhibited. See how the same fallacy leads to opposite results; the Erasistrateans thought that low fever was, like inflammatory fever, only the manifestation of a local lesion; the first is better *without venesection*, so therefore must be the latter; the Broussaists, the other day, re-advanced the same doctrine, but as the latter is better *for venesection*, blood was taken from both.

Thirdly. Do we not often succumb to popular prejudices against what our reason tells us is right? John Hunter himself used unfairly the typical expression of the Mosaic writings, about the blood being the life, and a great deal of the horror with which venesection is popularly regarded seems due to the Hebrew synonyme of "bloodshedding" for murder. Two days before the writing of this paragraph, a leading London physician, in stating to the writer his objection to bleeding, expressed himself as "*guiltless* of the blood of his patients."

Fourthly. Do not many of us commit the same error as Erasistratus, by thinking that the diminution of *quantity* is the only aim in taking blood? It is true that by starvation less will be made, and consequently that the total amount will be reduced. But then the reduction of quantity is only a temporary and partial result desired. The more important intention should be the improvement of *quality*, by removing some of the effete constituents destroyed by the disease, and so making room for as much fresh new blood as the system can furnish. Viewed in this light, bleeding can never have its place supplied by starvation; indeed, a sufficiency of nutriment is essential to its best success.

Let two things be remarked in the above quotation as to Galen's mode of educating his mind. First, that he allowed a few well observed and striking instances to influence him more than any general impression derived from the diluted experience of external practice. He may be right or wrong, but this is certainly his way of arguing, both with himself and readers. Secondly, he lays great stress on Nature's mode of cure, and knows he cannot err in imitating it. Without hesitation he must be pronounced right in this.

The specimen given of our author's easy writing is quite sufficient to explain why our limits do not allow us to exhibit the way in which, through eight more chapters, he descends in a cataract of words upon the elderly practitioners of the metropolis. This is not by any means the only example of his unbridled tongue; he himself tells us of his concluding an argument with ————————* which, however true, is unconciliating to an adversary; and it is not surprising that he soon made his adopted home too hot to hold him, and went back to Pergamos after four years' sojourn, only to return when sure of Imperial patronage and protection against the natural consequences of such phrases.

A few observations, however, of his in the latter chapters of the above quoted lecture must not be passed over. He says that loss of blood certainly does not increase the tendency to inflammation; because gladiators who bled much after incised wounds in the circus usually had their injuries healed by the first intention. On the contrary, it would appear that this unintentional venesection was a favorable occurrence. He makes also a pertinent remark on the danger of excessive abstinence—namely, that the stomach is apt to be so much weakened thereby, that food when afterwards administered is not digested. Again, he gives us a useful hint about the way in which the authorities of great names are sometimes quoted as having employed a particular line of practice, without any reference being made to the success, or the contrary, which attended it. Thus two instances accurately detailed by Erasistratus of the treatment of inflammation without bleeding were thrown in Galen's teeth by his opponents; but when they are looked into, behold, both are fatal! Dr. Markham has pointed out an instance of the same identical fallacy leading to an opposite result in our own day. Andral, led by the authority of his masters, advocates a practice of venesection justly called excessive. But when, with rare ingenuity, he details the cases in which it was practised, a mortality much above the average is made evident.

Another tract written by Galen, seemingly about the same time, is a more direct commentary on the arguments employed not so much by the disciples as the master; it is entitled, 'On Bleeding, against Erasistratus,' and he may fairly be considered to have given a sufficient answer to all the arguments brought forward, at least at that period, against the practice.

Having thus cleared the ground of those gainsayers who represented venesection as in itself objectionable under all circumstances, he lays

* See his treatise On the Presence of Blood in the Arteries. Edit. Kuhn, vol. iv. p. 728.

before the public his well known 'Therapeutical Essay on Phlebotomy.* He publishes this as an appendix to his lectures 'On the Preservation of health,' and 'On Rational Therapeutics,' not voluntarily, as he says at the end of the fifth chapter, but in consequence of external pressure; so that it may be looked upon in the light of an "Apology." It is a rich mine of suggestions, from whence authors have dug most freely, sometimes gold, and sometimes dross. So that it is rare to find any writing on the subject, where, acknowledged or unacknowledged, at first, second, or third hand, some scraps of the loquacious Greek do not appear.

A specimen may be here given of his mode of referring his argument to rational physiolgy, extracted from a chapter in this essay, explaining how the heart and other organs are affected by changes in the fluids. By translating his imperfect chemical nomenclature into the more accurate language of the present day, it is singular how closely he is made to approach the speculations of our cotemporaries; and one sighs in reading the first sentence to think he should have hovered so near the brink of discovering the circulation, which the world was obliged to do without for fifteen centuries more. The way in which anatomy, chemistry, and mechanics are, as handmaids to physiology, brought to bear on practice, is quite in the style of the best school of modern rational medicine, and is really a model to be followed.

"The blood not only supplies nutriment to the organism, but continuously keeps up the animal heat, just as a hot-air stove is kept alight with firewood, so that the whole house is warmed by it.† As, then, this fire is damped sometimes by too many logs being thrown on to it at once, sometimes not by their being in too great quantity, but by their wet, sometimes by none at all or too few being put on; so the heat derived from the circulation may be reduced below its natural standard either by the supply of blood exceeding the demand, or by the demand exceeding the supply, or by the incombustible quality of the blood. Or, on the other hand, it may be increased either by the

---

* Edit. Kuhn, vol. xi. p. 250.

† The Pompeian House at the Crystal Palace has misled many into contempt for a Roman's notion of making himself comfortable in cold weather. But a different tale is told by the elaborate warming apparatus in the basement of Constantine's Palace at Treves (all brick architects should visit Treves;) and the above illustration shows that the application of the "focus" to warm all the rooms in the house by tubes was common among all classes. There was only one stove in each tenement; for "focus" is used by Horace as a synonym for a country house; and by an economical arrangement, not only did that warm the apartments, but, as appears from Cato, was used also for Cooking (Cato de R. R., 10 and 11,) and for baking bread (id. 75.)

heating quality of the blood, or by the natural demand being absent. Now, whatever change takes place in the combustion at the centres of circulation is quickly communicated to the rest of the body.

"But occasionally, as I have often shown in other memoirs, some one organ alone is morbidly altered in temperature; and that may be referred to two sources—namely, sometimes to the heating or cooling nature of the fluids, sometimes to a morbid crasis only. Just in the same way the structure of the heart may be subjected to morbid changes in a demonstrable manner from two sources—namely, either from the combustibility or the contrary of the fluids, or from some deficiency in them.

"The quickness or slowness of this combustion has been demonstrated to depend on the amount of food and drink ingested, and on the amount of repose or activity of body and mind. But as in the abdominal viscera there often occurs imperfect digestion, the ingesta degenerating into mucus or bile, or undergoing some other morbid chemical change, or remaining entirely unaltered, and fermenting into flatus—just so when the blood-making functions are deficient there will occur peculiar states of the fluids in arteries and veins analogous to the above-named derangements of digestion in the abdomen.

"Now since the heat and moisture of a substance seems to augment the rapidity of its chemical decompositions, especially if it be in a warm place, it will be a necssary consequence of this principle, that the nutritive matters distributed to the system through the medium of the abdominal viscera should undergo somewhere or other some species of decomposition, whenever they have not that tendency arrested by being converted normally into healthy blood. But since the decompositions which take place in matters capable of supporting combustion, heighten the temperature, therefore the blood in which the above-described decomposition is going on will exhibit an abnormal degree of heat. . . . . . . When that high temperature is once generated, the whole body is easily heated by it, just as the whole house is by a great fire in the stove. This condition of system the Greeks call fever."

The rules given by Galen for the guidance of the practitioner are too diffuse for "our limits." Perhaps under the pressure of editorial scissors he might have condensed his thoughts into theses more suited to citation. However, the guiding principles seem to be:

1. That you are not to treat the disease but the man; that you are to judge of the propriety, the amount, and necessity for repetition of bloodletting, by the individual symptoms exhibited in each case, and not by the nomenclature.

2. That you are to observe, also, the natural constitution of the patient—e.g., the extremes of life, youth and old age, cause bloodletting to be badly borne. Certain races, such as the soft-fleshed Celtic nations,* do not stand it.

3. That you also take note of epidemic influences—e.g., not to bleed much in the dog days (in Italy ;) and in moist, warm weather, when of course septic poisons are most rife.

4. That you are not to mistake physiological changes for morbid; such, for example, as the fulness of pulse which accompanies the first stage of digestion, for permanent fulness.

5. That you are to take blood from vessels which communicate directly with the chief seat of inflammation.

6. That often, in spite of apparent or real general debility, it is desirable to take blood, since the benefit to the locally affected part, and the consequent benefit to the system, compensates for the depletion.

This rationalizing of Hippocrates' empirical practice of bleeding established it on a firm basis. So that, with the exception of an occasional "unhappy revival by some chemist" (Van Swieten,) the theories of Erasistratus have not again raised their heads; and the future history of the controversy has an interest more curious than practical. We will not attempt to follow it here, but will confine ourselves to the sketch given of one period, which, even if not the most critical, certainly has found the most spirited historian.—*British & Foreign Medico-Chirurgical Review.*

